# siDirect 2.0: updated software for designing functional siRNA with reduced seed-dependent off-target effect

**DOI:** 10.1186/1471-2105-10-392

**Published:** 2009-11-30

**Authors:** Yuki Naito, Jun Yoshimura, Shinichi Morishita, Kumiko Ui-Tei

**Affiliations:** 1 Department of Biophysics and Biochemistry, Graduate School of Science, University of Tokyo, 7-3-1 Hongo, Bunkyo-ku, Tokyo 113-0033, Japan; 2 Department of Computational Biology, Graduate School of Frontier Sciences, University of Tokyo, 5-1-5 Kashiwanoha, Kashiwa, Chiba 277-8562, Japan

## Abstract

**Background:**

RNA interference (RNAi), mediated by 21-nucleotide (nt)-length small interfering RNAs (siRNAs), is a powerful tool not only for studying gene function but also for therapeutic applications. RNAi, requiring perfect complementarity between the siRNA guide strand and the target mRNA, was believed to be extremely specific. However, a recent growing body of evidence has suggested that siRNA could down-regulate unintended genes whose transcripts possess complementarity to the 7-nt siRNA seed region. This off-target gene silencing may often provide incongruous results obtained from knockdown experiments, leading to misinterpretation. Thus, an efficient algorithm for designing functional siRNAs with minimal off-target effect based on the mechanistic features is considered of value.

**Results:**

We present siDirect 2.0, an update of our web-based software siDirect, which provides functional and off-target minimized siRNA design for mammalian RNAi. The previous version of our software designed functional siRNAs by considering the relationship between siRNA sequence and RNAi activity, and provided them along with the enumeration of potential off-target gene candidates by using a fast and sensitive homology search algorithm. In the new version, the siRNA design algorithm is extensively updated to eliminate off-target effects by reflecting our recent finding that the capability of siRNA to induce off-target effect is highly correlated to the thermodynamic stability, or the melting temperature (Tm), of the seed-target duplex, which is formed between the nucleotides positioned at 2-8 from the 5' end of the siRNA guide strand and its target mRNA. Selection of siRNAs with lower seed-target duplex stabilities (benchmark Tm < 21.5°C) followed by the elimination of unrelated transcripts with nearly perfect match should minimize the off-target effects.

**Conclusion:**

siDirect 2.0 provides functional, target-specific siRNA design with the updated algorithm which significantly reduces off-target silencing. When the candidate functional siRNAs could form seed-target duplexes with Tm values below 21.5°C, and their 19-nt regions spanning positions 2-20 of both strands have at least two mismatches to any other non-targeted transcripts, siDirect 2.0 can design at least one qualified siRNA for >94% of human mRNA sequences in RefSeq. siDirect 2.0 is available at http://siDirect2.RNAi.jp/.

## Background

RNA interference (RNAi) mediated by double-stranded RNA has become a powerful tool not only for studying gene functions, but also for therapeutic applications [1,2]. In mammalian cells, RNAi is induced by small interfering RNA (siRNA), a duplex of 21-nucleotide (nt) RNAs containing 2-nt 3' overhangs. The siRNAs incorporated into cells are transferred to the RNAi effector complex called RNA-induced silencing complex (RISC) [3,4]. RISC assembles on one of the two strands of siRNA duplex, and is activated upon the removal of the passenger strand [5-9]. The activated RISC is a ribonucleoprotein complex minimally consisting of the core protein Argonaute (Ago) and single-stranded siRNA, which acts as the guide to target complementary sequences within mRNAs [10-13]. The 5' end of the siRNA guide strand is anchored in the binding pocket of the Mid domain of *Archaeoglobus fulgidus *Ago-like protein [14,15], and the 3' end is anchored to the PAZ domain of human [16] and *Drosophila *[17] Ago in the RISC complex. Thus, in the siRNA guide strand, 19 nucleotides positioned at 2-20 from 5' end may be responsible for target RNA recognition, leading to the silencing of gene expression by cleaving target mRNA [10-13]. Since RNAi is based on sequence recognition by the siRNA, it can give rise to the silencing of other genes with similar sequences. This phenomenon is referred to as an off-target effect, and the growing evidence from large-scale knockdown experiments indicates that the off-target silencing is induced by the base-pairing between the seed region at positions 2-8 from the 5' end of the RISC-loaded siRNA strand, and its complementary sequences in the 3' UTR of the unrelated mRNAs [18-23]. Although RNAi is now widely and routinely used as an experimental tool, the remaining fundamental concern is whether the target gene can be specifically silenced. Especially, accurate knowledge of RNAi specificity is critical for therapeutic technologies.

To avoid off-target effects, one approach may be to select the siRNA whose seed sequence is not complementary to any sequences in the 3' UTR of all non-targeted genes. However, this approach is problematic because random 7-nt sequence is predicted to appear in every 16,384 bp on average. In fact, we analyzed the human 3' UTR database and it proved impossible to select such siRNAs. That is, human siRNAs with the most infrequent 7-nt seed sequence still have seed-complementarities with 17 3' UTR sequences. Recently, we have revealed that the capability of siRNAs to induce seed-dependent off-target effect is highly correlated to the thermodynamic stability of the duplex formed between the seed region of siRNA guide strand and its target mRNA [23]: the melting temperature (Tm) of the seed-target duplex showed strong positive correlation with the induction of seed-dependent off-target effects. The results suggested that the Tm of 21.5°C may serve as the benchmark, which discriminates the almost off-target-free seed sequences from the off-target-positive ones. Thus, selecting the siRNAs with low Tm of the seed-target duplex should minimize seed-dependent off-target silencing.

We have previously released highly effective, target-specific siRNA design software, siDirect [24], in which siRNA sequences were selected using our guidelines established through extensive experiments to clarify the relationship between siRNA sequences and RNAi activities [7]. In order to exclude potential cross-hybridization candidates, siDirect used the rigorous homology search algorithm to select siRNA sequences that have at least three mismatches to any other non-targeted transcripts [25]. In the updated software, siDirect 2.0, the siRNA design algorithm has been extensively updated to select off-target minimized siRNAs by considering the thermodynamic stability of the seed-target duplex. By using the default parameters, at least one functional siRNA could be designed for >94% of the human mRNA sequences in RefSeq release 30.

## Implementation

**Figure 1:**
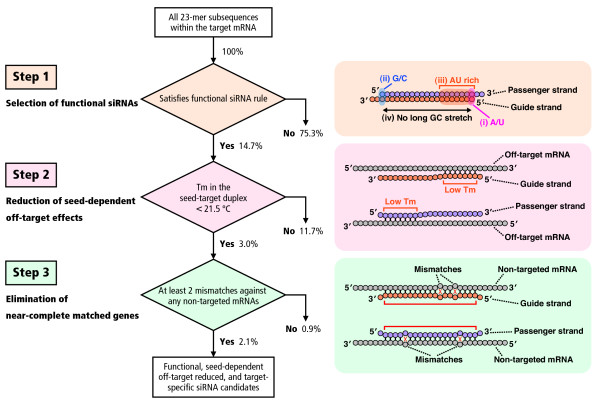
**Overall flow of siRNA selection in siDirect 2.0**. The functional and target-specific siRNAs were selected by three selection steps. In Step 1, functional siRNA sequences were selected according to our algorithm [7]. In Step 2, siRNAs with Tm values below 21.5°C in the seed-target duplex were selected. In Step 3, nucleotides positioned in the 2-20 of both strands of the siRNAs were subjected to the near-perfect match searches, and siRNAs that have at least two mismatches to any other non-targeted transcripts were selected. The percentages denote the proportions of selected ('Yes') or unselected ('No') siRNA candidates calculated using all 23-mer subsequences (56,375,087; 100%) generated from human mRNAs in RefSeq release 30.

Overall flow of siRNA selection in siDirect 2.0 is illustrated in Figure 1. All possible 23-mer subsequences, corresponding to the complementary sequence of 21-nt guide strand and 2-nt 3' overhang of the passenger strand within the target sequence, are generated and filtered in three selection steps described below.

### Selection of highly functional siRNAs

In the first step, highly functional siRNA sequences were selected using our algorithm [7] (Figure 1, Step 1). We have revealed that efficient RNAi could be induced by the siRNAs that satisfies the following three sequence conditions simultaneously: A/U at the 5' terminus of the guide strand; G/C at the 5' terminus of the passenger strand; at least 4 A/U residues in the 5' terminal 7 bp of the guide strand. In addition, G/C stretch longer than 9 bp should be absent [7]. The experimental validation showed that 98% of the siRNAs predicted to be functional have reduced the target gene expression [26]. The proportion of functional siRNA sequences selected by this algorithm is 14.7% of all human 23-mer sequences generated from RefSeq 30 (Figure 1A, see Step 1).

### Reduction of seed-dependent off-target effects

**Figure 2:**
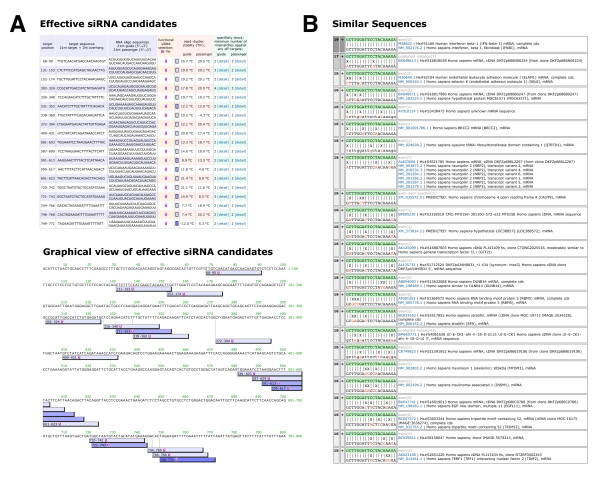
**Screenshots from siDirect 2.0 webserver**. **(A) **A typical output of siDirect 2.0: siRNAs targeting human interferon β-1 (NM_002176) are designed. **(B) **By clicking the individual siRNA in (A), a detailed list of off-target gene candidates with near-perfect matches is displayed separately for each siRNA strand. The alignment between each off-target sequence and the siRNA sequence clearly visualizes the positions of mismatches.

We have found that the off-target effect is highly correlated with the thermodynamic stability or Tm of the seed-target duplex, which is formed between the nucleotides positioned at 2-8 from the 5' end of the siRNA guide strand and its target sequence [23]. In the second step, to avoid off-target effect, Tm for the seed-target duplex was calculated using the nearest neighbor model and the thermodynamic parameters for the formation of RNA duplex as described previously [23] (Figure 1, Step 2). The formula for calculating Tm is: Tm = {(1000 × Δ*H*)/(*A *+ Δ*S *+ *R *ln(*C*_T_/4))} - 273.15 + 16.6 log [Na^+^], where Δ*H *(kcal/mol) is the sum of the nearest neighbor enthalpy change, *A *is the helix initiation constant (-10.8), Δ*S *is the sum of the nearest neighbor entropy change [27], *R *is the gas constant (1.987 cal/deg/mol), and *C*_T _is the total molecular concentration of the strand (100 μM). [Na^+^] was fixed at 100 mM. As shown in our previous report, calculated Tm of 21.5°C may be a benchmark to discriminate almost off-target-free seed sequences from the off-target-positive ones [23], and thus used as the initial standard in this study. Furthermore, it has been revealed that RNAi silencing is occasionally induced by the passenger strands of functional siRNAs [23], and that the passenger strands also take part in the seed-dependent off-target gene silencing [18,28]. Thus, siRNAs whose seed-target Tm is below 21.5°C for both guide and passenger strands were selected in this study. In consequence, 3.0% of all human 23-mer sequences remained available (Figure 1A, see Step 2). Calculated Tm value for each siRNA is shown in the siDirect 2.0 output page (Figure 2A).

### Elimination of near-perfect matched genes

Several studies have indicated that the effect of single-base mismatches between the siRNA guide strand and the target mRNA varies, according to the positions of the mismatch and/or the sequence of siRNA [21,29]. However, as shown in our previous report, it is obvious that even when the Tm value of the seed-target duplex is sufficiently low, the target gene silencing can still take place if the non-seed region is completely complementary [23]. Therefore, in the third step, siRNAs that have near-perfect matches to any other non-targeted transcripts were eliminated. In siDirect 2.0, off-target searches are performed for 19-mer sequences at positions 2-20 of both strands of the siRNA duplex (Figure 1B, Step 3), because these 19 nucleotides are thought to be involved in target mRNA recognition. Since widely-used BLAST tends to overlook near-perfect match candidates frequently, we used our fast and sensitive algorithm [25]. In addition, all of the near-perfect match hits are precomputed for all the functional human siRNAs to accelerate the computational performance. Precomputed results are stored in the memory engine of MySQL relational database management system. This makes it possible to return the list of siRNA candidates within a few seconds (Figure 2A). The output page includes the minimum number of mismatches against any near-perfect match candidates for each siRNA (Figure 2A). By clicking the individual siRNA in Figure 2A, a detailed list of candidate genes will appear (Figure 2B). By default, siRNA sequences that have at least two mismatches to any other non-targeted transcripts are selected.

## Results and Discussion

We performed a genome-wide design of siRNAs for human mRNAs in RefSeq release 30 with the following parameters: 1) satisfying our functional siRNA design algorithm [7,24], 2) Tm values at the seed-target duplex of both the guide and the passenger strands below 21.5°C, and 3) no off-target hits with less than two mismatches.

**Figure 3:**
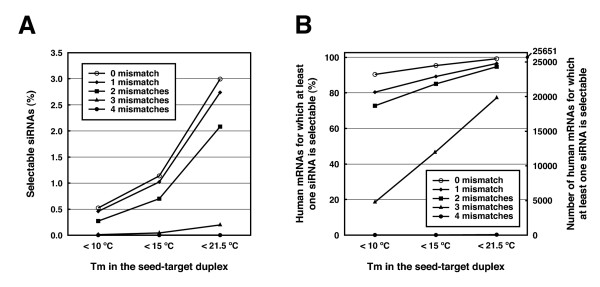
**The proportion of selectable siRNAs and mRNAs according to Tm values in the seed-target duplexes**. **(A) **The percentage of selectable siRNA candidates for human mRNAs according to the Tm values in the seed-target duplexes. The total number of siRNA (56,375,087) is set to 100%. **(B) **The percentage of human mRNAs harboring at least one target sequence of an siRNA whose Tm value of the seed-target duplex is below the indicated value. 100% indicates 25,651 mRNAs. Off-target hits with 0-4 mismatches between nucleotides at positions in the 2-20 of both siRNA strands and human mRNAs were represented as separate lines.

The degree of off-target effects is shown to be correlated with the thermodynamic stability or the calculated Tm value of the seed-target duplex [23]. The initial boundary Tm value was set to 21.5°C to discriminate the off-target-free sequences from the off-target-positive ones, according to our previous report [23]. Among the entire siRNA sequence population that have at least two mismatches to any other non-targeted transcripts, the siRNA sequences with seed-target Tm below 21.5°C account for 2.1% of about 56 million 23-mer fragments found in human mRNAs (Figure 3A), and one or more siRNA can be designed for 94.7% of all human mRNAs (Figure 3B). However, the strong correlation between the calculated Tm and the off-target gene silencing activity indicates that the seed-dependent off-target effect is definitively reduced when the siRNA with lower Tm of seed-target duplex are selected. The population of siRNAs among all human 23-mer sequences with the Tm in the seed-target duplex of less than 15°C and 10°C is 0.7% and 0.3%, respectively (Figure 2A), and the fraction of human mRNAs which can be targeted by more than one siRNA within such criteria decreases to 85.1% and 72.7%, respectively. (Figure 3B).

It is also desirable to select siRNA that contains as many mismatches as possible to any non-targeted mRNAs. In addition to the Tm value of below 21.5°C, siRNA sequences with at least two mismatches to any other non-targeted transcripts are selectable for 94.7% of human mRNAs (Figure 3B). However, if the siRNAs having near-perfect match hits with less than three mismatches, with their Tm of seed sequences below 21.5°C, are selected, one or more siRNA can be designed for only 77.2% of the human mRNAs (Figure 3B). When siRNAs with seed Tm below 15°C and 10°C were selected, siRNAs can be designed for only 47.0% and 18.5%, respectively (Figure 3B). Furthermore, the percentage of human mRNAs drops severely to 0.15% if the near-perfect match hits with less than four mismatches are filtered. Thus, siDirect 2.0 filters siRNAs with less than two mismatches by default to avoid severe reduction in the number of siRNA candidates.

We were unable to design functional, off-target minimized siRNAs for 5.3% of the RefSeq mRNAs using the default parameters. Typical examples of these mRNAs are the histone clusters (NM_003523, etc.) and ribosomal proteins (NM_002952, etc.), which are known to form multigene families. When designing siRNAs targeting such genes, users can manually investigate the detailed list of off-target gene candidates (Figure 2B) and select the siRNA that does not have off-target hits to unrelated transcripts.

**Figure 4:**
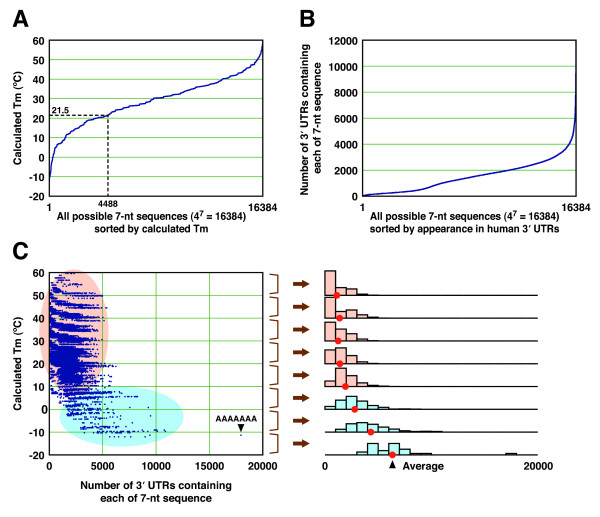
**Calculated Tm values and appearances of 7-nt seed sequences**. **(A) **Calculated Tm values of the duplex formed by all possible 7-nt sequences. The dotted line indicates that the number of 7-nt sequences with duplex Tm below 21.5°C is 4,488 (27.4%). **(B) **Appearance of 7-nt seed sequences in human 3' UTRs. The numbers of 3' UTR sequences containing at least one given 7-nt sequence are shown. **(C) **Relationship between the appearance of each 7-nt sequence in the 3' UTRs containing at least one 7-nt sequence and its calculated Tm. Histograms in the right panel show the appearance of each 7-nt sequence in human 3' UTR, divided into 10°C Tm intervals. The seed sequence whose duplex has lower Tm (colored blue) is more frequently observed in the 3' UTRs as compared to those with higher Tm (colored orange).

Although most existing web servers for designing siRNA incorporate BLAST [30] to avoid off-target effects [31-38], several sites including WI siRNA Selection Server [34], siDRM [39], DSIR [40] and Dharmacon siDESIGN Center consider seed-dependent off-target effects. Current version of WI siRNA Selection Server and siDRM enumerates the transcripts with full homology to the seed region, and DSIR and Dharmacon siDESIGN Center calculate seed frequencies for each siRNA candidate. Therefore, we analyzed the relationship between the calculated Tm and the distribution of each seed sequence in human 3' UTRs. Calculated Tm of the seed-target duplexes of all possible 7-nt seed sequences (4^7 ^= 16,384) ranged from -12°C to 60°C, and of these, 4488 (27.4%) 7-mers had the Tm below 21.5°C (Figure 4A). The number of 3' UTRs bearing at least one target site of any 7-nt sequence was broadly distributed from 17 to 10,882 (Figure 4B), excluding the sequence AAAAAAA, which is found in almost all 3' UTRs with poly(A) tails. When the siRNAs were classified into eight groups according to their Tm of the seed-target duplex, as shown in Figure 4C, siRNAs whose seed-target duplexes had higher Tm, ranging from 20°C to 60°C, were less frequent and similarly distributed. On the other hand, the seed sequences with lower Tm were frequently found in human 3' UTRs (Figure 4C).

## Conclusion

We have extensively updated siDirect 2.0 based on our experimental knowledge, and provided a promising website for reducing siRNA off-target silencing. The website selects: 1) functional siRNAs that satisfy our guideline [7], 2) siRNAs with reduced seed-dependent off-target effects by considering the thermodynamic stability of the seed-target duplex, 3) siRNAs that do not hit any non-targeted genes with near-perfect matches. When the candidate functional siRNAs could form seed-target duplexes with Tm values below 21.5°C, and their 19-nt region spanning positions 2-20 of both strands have at least two mismatches to any other non-targeted transcripts, siDirect 2.0 can design at least one qualified siRNA for >94% of human mRNA sequences in RefSeq. This website should provide a wide scope of applications in RNAi studies.

## Availability and requirements

Project name: siDirect

Project home page: http://siDirect2.RNAi.jp/

Operating system(s): Platform independent

Programming language: Perl

Any restrictions to use by non-academics: Contact license@RNAi.jp

## Authors' contributions

YN developed the webserver and performed the computational analyses. JY and SM developed the core framework of the software. KU-T supervised the entire study. YN and KU-T drafted the manuscript. All authors read and approved the final manuscript.

## References

[B1] Boutros M Ahringer J The art and design of genetic screens: RNA interference Nat Rev Genet 2008 9 554 566 10.1038/nrg2364 18521077

[B2] Castanotto D Rossi JJ The promises and pitfalls of RNA-interference-based therapeutics Nature 2009 457 426 433 19158789 10.1038/nature07758 PMC2702667

[B3] Hutvagner G Simard MJ Argonaute proteins: key players in RNA silencing Nat Rev Mol Cell Biol 2008 9 22 32 10.1038/nrm2321 18073770

[B4] Jinek M Doudna JA A three-dimensional view of the molecular machinery of RNA interference Nature 2009 457 405 412 10.1038/nature07755 19158786

[B5] Schwarz DS Hutvágner G Du T Xu Z Aronin N Zamore PD Asymmetry in the assembly of the RNAi enzyme complex Cell 2003 115 199 208 10.1016/S0092-8674(03)00759-1 14567917

[B6] Khvorova A Reynolds A Jayasena SD Functional siRNAs and miRNAs exhibit strand bias Cell 2003 115 209 216 10.1016/S0092-8674(03)00801-8 14567918

[B7] Ui-Tei K Naito Y Takahashi F Haraguchi T Ohki-Hamazaki H Juni A Ueda R Saigo K Guidelines for the selection of highly effective siRNA sequences for mammalian and chick RNA interference Nucleic Acids Res 2004 32 936 948 14769950 10.1093/nar/gkh247 PMC373388

[B8] Matranga C Tomari Y Shin C Bartel DP Zamore PD Passenger-strand cleavage facilitates assembly of siRNA into Ago2-containing RNAi enzyme complexes Cell 2005 123 607 620 10.1016/j.cell.2005.08.044 16271386

[B9] Rand TA Petersen S Du F Wang X Argonaute2 cleaves the anti-guide strand of siRNA during RISC activation Cell 2005 123 621 629 10.1016/j.cell.2005.10.020 16271385

[B10] Elbashir SM Lendeckel W Tuschl T RNA interference is mediated by 21- and 22-nucleotide RNAs Genes Dev 2001 15 188 200 11157775 10.1101/gad.862301 PMC312613

[B11] Meister G Landthaler M Patkaniowska A Dorsett Y Teng G Tuschl T Human Argonaute2 mediates RNA cleavage targeted by miRNAs and siRNAs Mol Cell 2004 15 185 197 10.1016/j.molcel.2004.07.007 15260970

[B12] Song JJ Smith SK Hannon GJ Joshua-Tor L Crystal structure of Argonaute and its implications for RISC slicer activity Science 2004 305 1434 1437 10.1126/science.1102514 15284453

[B13] Liu J Carmell MA Rivas FV Marsden CG Thomson JM Song JJ Hammond SM Joshua-Tor L Hannon GJ Argonaute2 is the catalytic engine of mammalian RNAi Science 2004 305 1437 1441 10.1126/science.1102513 15284456

[B14] Parker JS Roe SM Barford D Structural insights into mRNA recognition from a PIWI domain-siRNA guide complex Nature 2005 434 663 666 10.1038/nature03462 15800628 PMC2938470

[B15] Ma J-B Yuan YR Meister G Pei Y Tuschl T Patel DJ Structural basis for 5'-end-specific recognition of guide RNA by the *A. fulgidus *Piwi protein Nature 2005 434 666 670 10.1038/nature03514 15800629 PMC4694588

[B16] Ma J-B Ye K Patel DJ Structural basis for overhang-specific small interfering RNA recognition by the PAZ domain Nature 2004 429 318 322 10.1038/nature02519 15152257 PMC4700412

[B17] Lingel A Simon B Izaurralde E Sattler M Nucleic acid 3'-end recognition by the Argonaute2 PAZ domain Nat Struct Mol Biol 2004 11 576 577 10.1038/nsmb777 15156196

[B18] Jackson AL Bartz SR Schelter J Kobayashi SV Burchard J Mao M Li B Cavet G Linsley PS Expression profiling reveals off-target gene regulation by RNAi Nat Biotechnol 2003 21 635 637 10.1038/nbt831 12754523

[B19] Scacheri PC Rozenblatt-Rosen O Caplen NJ Wolfsberg TG Umayam L Lee JC Hughes CM Shanmugam KS Bhattacharjee A Meyerson M Collins FS Short interfering RNAs can induce unexpected and divergent changes in the levels of untargeted proteins in mammalian cells Proc Natl Acad Sci USA 2004 101 1892 1897 14769924 10.1073/pnas.0308698100 PMC357023

[B20] Lin X Ruan X Anderson MG McDowell JA Kroeger PE Fesik SW Shen Y siRNA-mediated off-target gene silencing triggered by a 7 nt complementation Nucleic Acids Res 2005 33 4527 4535 16091630 10.1093/nar/gki762 PMC1184219

[B21] Birmingham A Anderson EM Reynolds A Ilsley-Tyree D Leake D Fedorov Y Baskerville S Maksimova E Robinson K Karpilow J Marshall WS Khvorova A 3' UTR seed matches, but not overall identity, are associated with RNAi off-targets Nat Methods 2006 3 199 204 10.1038/nmeth854 16489337

[B22] Jackson AL Burchard J Schelter J Chau BN Cleary M Lim L Linsley PS Widespread siRNA "off-target" transcript silencing mediated by seed region sequence complementarity RNA 2006 12 1179 1187 16682560 10.1261/rna.25706 PMC1484447

[B23] Ui-Tei K Naito Y Nishi K Juni A Saigo K Thermodynamic stability and Watson-Crick base pairing in the seed duplex are major determinants of the efficiency of the siRNA-based off-target effect Nucleic Acids Res 2008 36 7100 7109 18988625 10.1093/nar/gkn902 PMC2602766

[B24] Naito Y Yamada T Ui-Tei K Morishita S Saigo K siDirect: highly effective, target-specific siRNA design software for mammalian RNA interference Nucleic Acids Res 2004 32 W124 W129 15215364 10.1093/nar/gkh442 PMC441580

[B25] Yamada T Morishita S Accelerated off-target search algorithm for siRNA Bioinformatics 2005 21 1316 1324 10.1093/bioinformatics/bti155 15564304

[B26] Naito Y Saigo K Ui-Tei K Lyland RT, Browning IB Evaluation of published rational siRNA design algorithms using firefly *luciferase *gene as a reporter RNA interference research progress 2008 New York: Nova Science Publishers 3 11

[B27] Freier SM Kierzek R Jaeger JA Sugimoto N Caruthers MH Neilson T Turner DH Improved free-energy parameters for predictions of RNA duplex stability Proc Natl Acad Sci USA 1986 83 9373 9377 2432595 10.1073/pnas.83.24.9373 PMC387140

[B28] Clark PR Pober JS Kluger MS Knockdown of TNFR1 by the sense strand of an ICAM-1 siRNA: dissection of an off-target effect Nucleic Acids Res 2008 36 1081 1097 18096615 10.1093/nar/gkm630 PMC2275081

[B29] Du Q Thonberg H Wang J Wahlestedt C Liang Z A systematic analysis of the silencing effects of an active siRNA at all single-nucleotide mismatched target sites Nucleic Acids Res 2005 33 1671 1677 15781493 10.1093/nar/gki312 PMC1069010

[B30] Altschul SF Gish W Miller W Myers EW Lipman DJ Basic local alignment search tool J Mol Biol 1990 215 403 410 2231712 10.1016/S0022-2836(05)80360-2

[B31] Levenkova N Gu Q Rux JJ Gene specific siRNA selector Bioinformatics 2004 20 430 432 10.1093/bioinformatics/btg437 14960474

[B32] Chalk AM Wahlestedt C Sonnhammer EL Improved and automated prediction of effective siRNA Biochem Biophys Res Commun 2004 319 264 274 10.1016/j.bbrc.2004.04.181 15158471

[B33] Henschel A Buchholz F Habermann B DEQOR: a web-based tool for the design and quality control of siRNAs Nucleic Acids Res 2004 32 W113 W120 15215362 10.1093/nar/gkh408 PMC441546

[B34] Yuan B Latek R Hossbach M Tuschl T Lewitter F siRNA Selection Server: an automated siRNA oligonucleotide prediction server Nucleic Acids Res 2004 32 W130 W134 15215365 10.1093/nar/gkh366 PMC441504

[B35] Santoyo J Vaquerizas JM Dopazo J Highly specific and accurate selection of siRNAs for high-throughput functional assays Bioinformatics 2005 21 1376 1382 10.1093/bioinformatics/bti196 15591357

[B36] Shah JK Garner HR White MA Shames DS Minna JD sIR: siRNA Information Resource, a web-based tool for siRNA sequence design and analysis and an open access siRNA database BMC Bioinformatics 2007 8 178 17540034 10.1186/1471-2105-8-178 PMC1896181

[B37] Chalk AM Sonnhammer EL siRNA specificity searching incorporating mismatch tolerance data Bioinformatics 2008 24 1316 1317 10.1093/bioinformatics/btn121 18397893

[B38] Park YK Park SM Choi YC Lee D Won M Kim YJ AsiDesigner: exon-based siRNA design server considering alternative splicing Nucleic Acids Res 2008 36 W97 W103 18480122 10.1093/nar/gkn280 PMC2447810

[B39] Gong W Ren Y Zhou H Wang Y Kang S Li T siDRM: an effective and generally applicable online siRNA design tool Bioinformatics 2008 24 2405 2406 10.1093/bioinformatics/btn442 18718944

[B40] Vert JP Foveau N Lajaunie C Vandenbrouck Y An accurate and interpretable model for siRNA efficacy prediction BMC Bioinformatics 2006 7 520 17137497 10.1186/1471-2105-7-520 PMC1698581

